# Protein innovation through template switching in the *Saccharomyces cerevisiae* lineage

**DOI:** 10.1038/s41598-021-01736-y

**Published:** 2021-11-19

**Authors:** May Abraham, Einat Hazkani-Covo

**Affiliations:** Department of Natural and Life Sciences, The Open University of Israel, Ra’anana, Israel

**Keywords:** Evolutionary genetics, Molecular evolution

## Abstract

DNA polymerase template switching between short, non-identical inverted repeats (IRs) is a genetic mechanism that leads to the homogenization of IR arms and to IR spacer inversion, which cause multinucleotide mutations (MNMs). It is unknown if and how template switching affects gene evolution. In this study, we performed a phylogenetic analysis to determine the effect of template switching between IR arms on coding DNA of *Saccharomyces cerevisiae*. To achieve this, perfect IRs that co-occurred with MNMs between a strain and its parental node were identified in *S. cerevisiae* strains. We determined that template switching introduced MNMs into 39 protein-coding genes through *S. cerevisiae* evolution, resulting in both arm homogenization and inversion of the IR spacer. These events in turn resulted in nonsynonymous substitutions and up to five neighboring amino acid replacements in a single gene. The study demonstrates that template switching is a powerful generator of multiple substitutions within codons. Additionally, some template switching events occurred more than once during *S. cerevisiae* evolution. Our findings suggest that template switching constitutes a general mutagenic mechanism that results in both nonsynonymous substitutions and parallel evolution, which are traditionally considered as evidence for positive selection, without the need for adaptive explanations.

## Introduction

Inverted repeats (IRs) are sequences with two copies of a DNA sequence in a reverse-complement orientation (e.g., 5′ATGTGxxxxCACAT 3′). IRs include internal symmetry, enabling them to switch between inter-strand and intra-strand base-pairing, resulting in non-canonical DNA structures such as cruciforms and hairpins. Long IRs lead to genome instability^1,2^, either because they are processed to create a double-strand break or because they block the replication fork^1–6^. The resulting genomic instability events are diverse, and include gene amplification^7–9^, translocations^10–13^, insertions^14^, and deletions^2^. Despite the potential of IRs to destabilize genomes, short IRs have several functions in organisms throughout the tree of life, e.g., IRs found in promoters enable the binding of homodimer transcription factors^15^. IRs also have a functional role in the viral origin of replications^16^, the CRISPR immune system^17^, alternative termination of bacterial genes^18^, and immunoglobulin V(D)J rearrangement^19^.

A striking characteristic of IRs is their tendency to undergo homogenization, abolishing variation between the two arms of the IR. Numerous examples have been reported of long-IR^20–25^ and short-IR homogenization^26–28^. From a mechanistic standpoint, DNA polymerase template switching can eliminate variations as short as a few bases between IR arms^28^. This occurs when one arm of the IR serves as a template for the synthesis of the second arm. Template switching, first suggested by Ripley (1982) in bacteriophages, requires two hops of DNA polymerase between templates and can occur either intramolecularly or intermolecularly^26,29–33^.

Since its identification, template switching has been reported throughout the tree of life^33–37^. Short-IR homogenization via template switching is known to occur in numerous organisms^26,27,35,36^ and is associated with mutational hotspots^27,34^. The extent of template switching is affected by the directionality of the replication fork^38–40^, the level of transcription^40^, and the local sequence context^41^. There are several genotypes in which template switching is more common. For example, we have previously shown that template switching occurs in yeast strains lacking Rad27p, a key player in Okazaki fragment maturation^42^.

While the evolutionary consequences of homogenization of long IRs are well-studied^21,43–47^, the impact of short IR homogenization on genome evolution has received little attention. Our previous evolutionary analysis of non-coding regions with short IRs in proteobacteria orthologs^48^ indicated that these regions are more conserved than their immediate surrounding. This suggests that repeated template switching between IR arms is common during the evolution of proteobacteria. Template switching between IR arms was also recently shown to be abundant in humans^49,50^.

To date there have only been a few reports of template switching in genes and these have usually been considered in their mutagenic context. Template switching was identified with the context of loss-of-function in T4 rII gene in T4^51^, *E. coli* rpsL^52^, and thyA^34^ genes, as well as in the *S. cerevisiae CYC1*^35^ gene. It was suggested that template switching contributes to the spectrum of mutations that affect the TP53 gene in human cancers^36^. Template switching was also shown to be involved in certain mutagenic processes that lead to several genetic diseases, such as hereditary angioneurotic edema^37^. In our previous analysis of *rad27* mutants in *S. cerevisiae*, we identified nine template switching events in coding genes^42^. It is unknown if and how template switching affects genes during evolution. The present work studied the effect of template switching on coding regions from an evolutionary perspective.

Template switching between short IR arms can cause mutation clusters though arm homogenization^53^. Multinucleotide mutations (MNMs), which comprise ~ 1% of single nucleotide polymorphisms in genomes^54–57^, form one type of mutation cluster, in which mutations appear at adjacent sites. MNMs in codons can be the outcome of two entirely different scenarios: mutational mechanisms that simultaneously affect nearby nucleotides or multiple changes that occur via adaptive evolution. Ignoring the contribution of mechanisms that simultaneously affect nearby nucleotides in codons may lead to false identifications of positive selection^54,55,57^. This is because positive selection tests determine nonsynonymous to synonymous ratios, while assuming an independency of mutations. Identifying the mechanisms that cause nonsynonymous replacements through MNMs is essential to the understanding of protein evolution. Template switching between short IR arms is a potential mechanism of MNM formation in genes.

Here, protein-coding genes of 50 closely related wild type *Saccharomyces cerevisiae* strains were analyzed to identify MNMs arising from template switching between IR arms. Such events were identified in 39 yeast proteins and were responsible for nonsynonymous substitutions and, thus, for amino acid replacements. While template switching primarily introduced single amino acid changes, events simultaneously affecting up to five nearby amino acids were also recorded. The presented results indicate that template switching is an important mechanism in protein evolution.

## Results

### IRs are associated with MNMs on IR arms in *wild type* yeast

**Figure 1:**
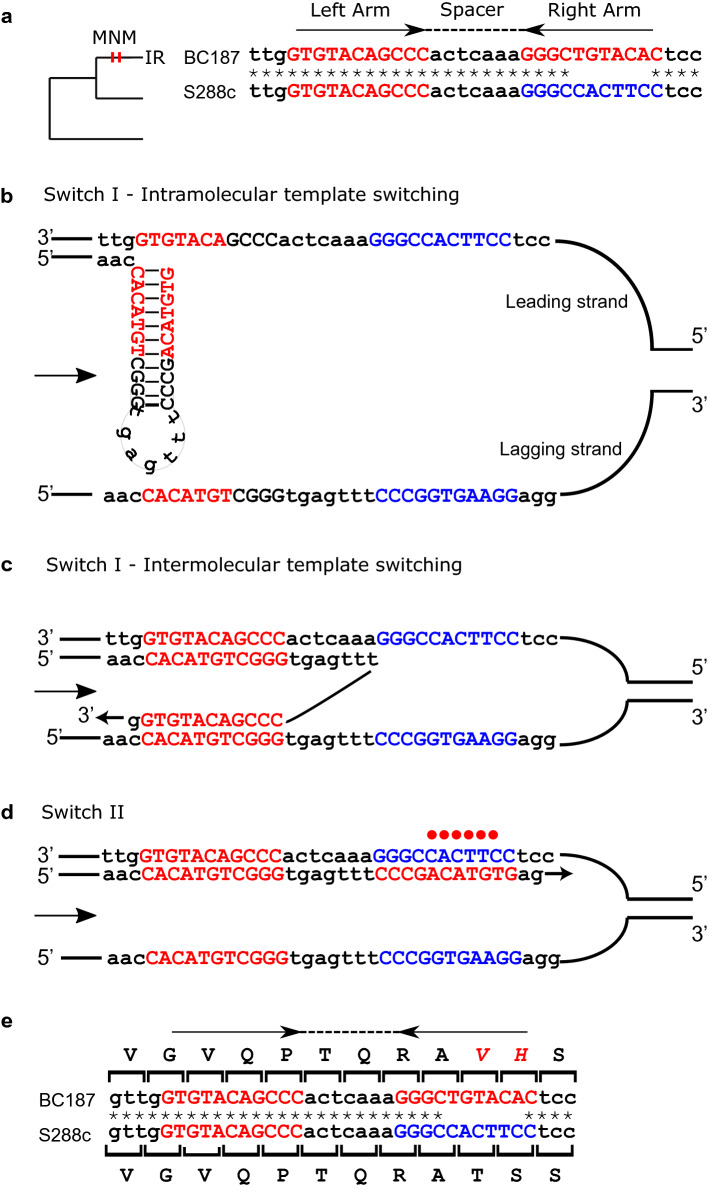
Template switching causes IR arm homogenization. (**a**) A recently occurring perfect IR in *UTP5* overlapped with a MNM on the branch leading to BC187 (shown as vertical red lines on the branch). Alignment of BC187 (perfect IR) and S288c (imperfect IR) is shown, with arrows representing IR arms, the dotted line representing the IR spacer and asterisks representing matches between strains. A very short IR with an arm length of 4 bp and a spacer of 7 bp appears in S288c. In strain BC187, a longer IR with an arm length of 11 bp was formed. (**b**–**d**) Template switching converted an imperfect IR to a perfect IR. (**b**) Assuming intramolecular template switching, the nascent strand folded on itself and served as a template. (**c**) Assuming intermolecular template switching, the nascent strand invades the sister chromatid and uses it as a template. (**d**) The second switch returns the nascent strand into the original template, resulting in a perfect IR, represented by the upper sequence in A. Uppercase letters represent the IR arms, with the same arms colored in the same color. The red dots represent mismatches between the arms. The direction of the replication fork is indicated by an arrow. (**e**) Six substitutions were induced during the template switching, resulting in the replacement of two amino acids (T38V, S39H).

To identify the effect of template switching between IR arms on coding genes, we sought out IRs associated with MNMs. To classify IRs as associated with MNMs, we first identified perfect IRs in a *S. cerevisiae* strains. Next, based on the reconstructed phylogenic tree, we identified MNMs that occurred between a strain and its parental node (see “Methods” section). Finally, we looked for cases of MNMs with coordinates overlapping IR arms that mapped to the terminal branch leading to the same strain with the IR (Fig. 1a). Identification of MNMs on the specific branch is based on ancestral sequence reconstruction. To increase the reliability of the analysis and avoid uncertainty resulting from ancestral sequence reconstruction, focus was placed on MNMs associated with IRs on external branches only. IRs with an arm length of ≥ 7 bp and a spacer ≤ 70 that are associated with MNMs were identified in 68 genes (Table 1).

**Table 1:** Number of genes that include IRs with an arm length of ≥ 7 bp and are associated with multinucleotide mutations.

Arm length	Number of genes with MNMs on arms only	Number of genes with a significant IR score compared to 100 random simulations (%)
16	1	*n.d.*
11	2	2 (100%)
10	2	2 (100%)
9	6	6 (100%)
8	16^a^	11 (69%)
7	42^a^	8 (20%)

^a^One gene had multinucleotide mutations **(**MNMs) in both inverted repeats (IRs), which were 7 bp and 8 bp long.

*n.d.* not detected.

Next, we sought to determine whether the identified events differ from what could be expected when no special mechanism for IR homogenization exists. If no specific mechanism acts on IRs, then IR regions are expected to evolve under the same mutation-selection regime as non-IR regions in the gene. To test the significance of the association between IRs and MNMs in *S. cerevisiae* genes and whether they can be ascribed to template switching between IR arms, we simulated each of the gene multiple sequence alignments (MSAs) until reaching 100 simulations with a similar number of IRs in the real data. All evolutionary parameters used for the simulations mirrored those of the real gene MSA: tree topology, branch lengths, phylogeny model, and proportion of invariant positions. An IR score was computed for each gene and each simulation, and represented the enrichment of MNMs presumably formed by IR homogenization. To account for MNM variation not associated with IRs, the score was calculated by dividing the number of IRs associated with MNMs by the number of non-IRs associated with MNMs (see “Methods” section). An IR score was calculated for each of the real genes and its 100 simulations. The empirical distribution of IR scores in 100 simulations served as a null distribution to which the score of the real gene was compared. If the value of the real IR score fell within 5% of the values of the IR scores of its 100 simulations, a gene was considered to have more MNMs associated with IRs than its simulations. Genes with a statistically significant association between MNMs and IRs were considered to have undergone template switching.

Our simulation revealed that IRs were significantly associated with MNMs in 30 out of 68 yeast genes (Table 1, Supplementary Tables 1, 2). The longer the IR arm, the higher the fraction of genes with a significant real IR score. For IRs with an arm length of 7 bp, only 19% (8/42) of the genes had a higher IR score than their null distribution; while for IRs with an arm length of 8 bp, 69% of the genes (11/16) had a higher IR score than their null distribution. All IRs with arm lengths of 9–11 bp (10/10) had a higher IR score than randomly expected (Table 1). Due to the inability to simulate sufficient sequences with an IR arm of length of 16 bp, no statistical evaluation was performed for this event. However, since the association between IR arm length and recent MNMs was stronger for longer IR arms, the association of a MNM with IR arm length 16 bp is likely real. In conclusion, template switching between IR arms formed MNMs and modified protein-coding DNA during the evolution of 30 *S. cerevisiae* genes.

The gene *UTP5* in BC187 *S. cerevisiae* strain has a perfect IR with an arm length of 11 bp, while at this locus S228c and other *S. cerevisiae* strains have an imperfect IR, with a continuous arm length of only 4 bp (Fig. 1b). Herein, we describe the template switching event that formed the perfect IR in BC187 from the ancestral form presenting in S288c (Fig. 1b–d). The process was comprised of two switches. The first switch moved the polymerase from its nascent template to the other IR arm, via either an intramolecular or intermolecular mechanism. In case of the intramolecular mechanism (Fig. 1b), the nascent strand folds upon itself using the arm base pairing. Thus the first arm is used as a template for replicating the second arm. In case of the intermolecular mechanism (Fig. 1c), the first switch is achieved when the nascent strand replicating one arm invades the template of the other sister chromatid. In both scenarios, the first switch is followed by a second switch, whereupon the polymerase returns to use the original strand as a template (Fig. 1d). The fork then resolves, leaving one daughter cell with an imperfect IR and one daughter cell with a perfect IR. The template switching in *UTP5* resulted in six base substitutions and two amino acid replacements (T38V, S39H) in strain BC187 (Fig. 1e).

**Figure 2:**
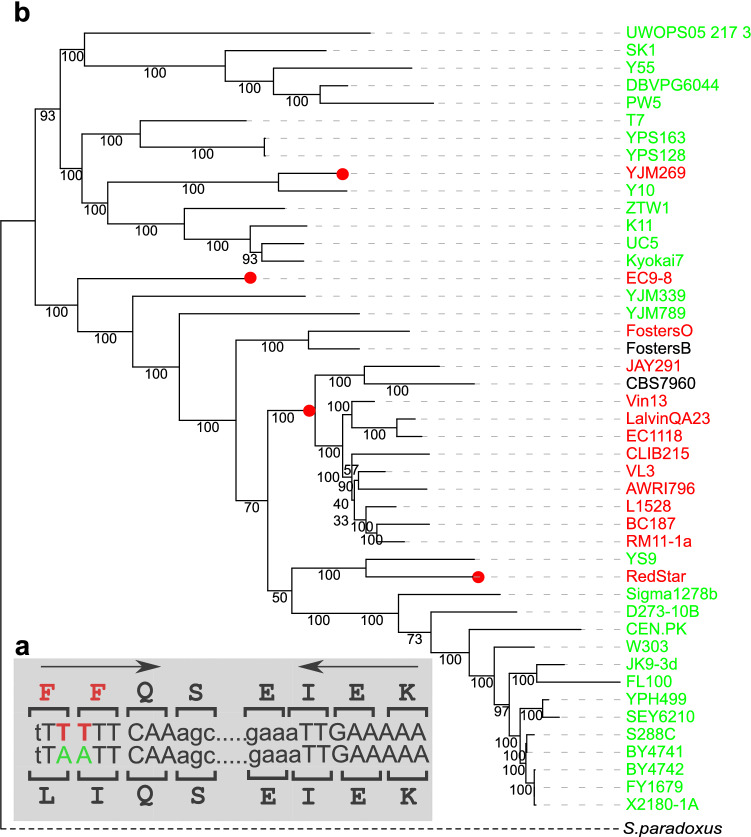
Template switching events forming an IR in the gene *MSH4*. (**a**) The homogenous IR has an arm length of 8 bp (upper sequence). The IR homogenesis was formed by an AA → TT MNM and resulted in two amino acid changes, i.e., L394F, I395F. The spacer is not shown. (**b**) Placing of template switching events on the tree. Fourteen strains with perfect IR and the TT form are shown in red, strains with the AA form are shown in green and other forms are shown in black. Template switching occurred on three terminal branches in *S. cerevisiae* lineage (red circles): on the branches leading to YJM269, RedStar, and EC9-8. While not directly estimated in this study, an ancestral sequence reconstruction revealed one extra event on one internal branch. The best maximum likelihood tree reconstructed by RAxML using DNA sequences from 4304 orthologs and the ancestral sequence reconstruction of codons 394–395 performed by FASTML are presented. (see “Methods” section). Bootstrap values are shown on branches. Tree outgroup is not drawn to scale.

Out of the 30 genes with IRs significantly associated with MNMs, 17 occurred uniquely in a single strain. The additional 13 genes included IRs that appeared in more than one *S. cerevisiae* strain, of which six had multiple IRs associated with MNMs on terminal branches (Supplementary Table 1). For example, in 14 strains, the *MSH4* gene had an IR with an arm length of 8 bp formed by an AA → TT MNM, which resulted in two amino acid changes, L394F and I395F (Fig. 2a). IR homogenization arose on three external branches of the *S. cerevisiae* phylogeny, leading to the strains YJM269, RedStar, and EC9-8. The ancestral sequence reconstruction revealed that one event had also occurred on one internal branch.

MNMs on IR arms formed by template switching caused nonsynonymous substitutions in 28 out of 30 genes. Twenty-five of these led to a single amino acid replacement, while two amino acid changes were observed in three genes. Thus, template switching is a source of parallel events and genetic innovation in proteins.

### Template switching causes spacer inversions and parallel evolution

**Figure 3:**
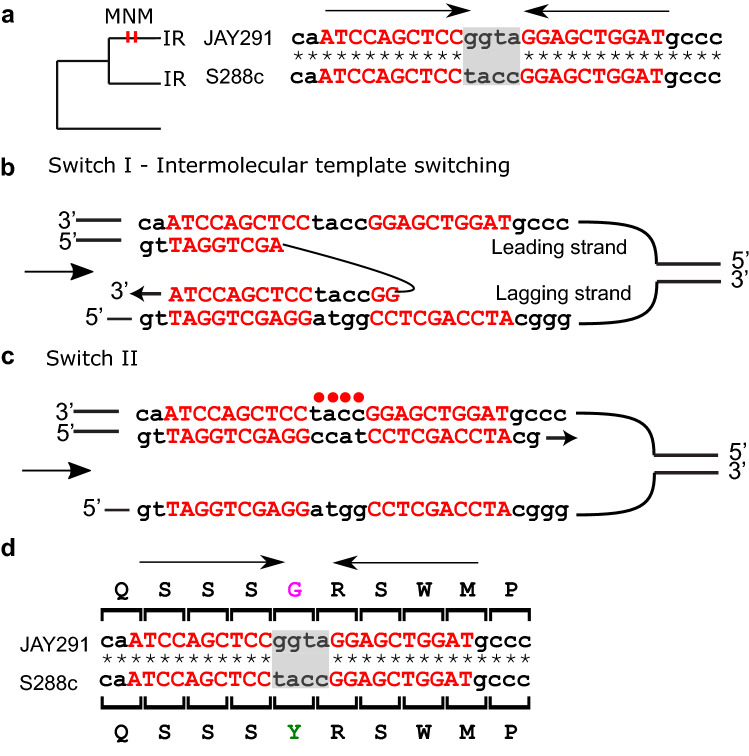
Intermolecular template switching causes spacer inversion. (**a**) A perfect IR in JAY291 on *SYG1* overlapping with a MNM located on the spacer. The MNM occurred in the branch leading to JAY291 (shown as vertical lines on the tree). In this case, the sister taxon, represented here by S288c, also has a perfect IR with the same arms. (**b**) The mechanism that created spacer inversion (AAGTC → GACTT) was intermolecular template switching, with the first switch having occurred during synthesis of the first arm. (**c**) The second switch returned the nascent strand to the original template, resulting in an inverted spacer of JAY291 compared to S288c. (**d**) IR spacer inversion resulted in one amino acid replacement (Y46G) in JAY291. Uppercase letters represent the IR arms, with the same arms shown in the same color. The red dots represent mismatches. The direction of the replication fork is indicated by an arrow.

Template switching between IR arms not only homogenizes the arms, but can form inversion of IR spacers^58,59^. This occurs when the first switch takes place through intermolecular template switching, during the replication of the first arm (Fig. 3). A search for IRs on *S. cerevisiae* strains carrying MNMs on the spacer, identified cases in which the MNM on the spacer arose from a complete spacer inversion. Ten IR spacer inversion events were identified within the coding sequence of *wild type* yeasts (Table 2, Supplementary Table 3). All events were statistically significant—none of the 100 simulations of the genes with spacer inversion showed inversions. Spacer inversions were observed only on IRs with arms longer than 9 bp. Inverted spacers ranged between 2 and 5 bp substitutions and resulted in up to two amino acid replacements.

**Table 2:** Genes with inverted repeats and spacer inversion.

Gene	IR arm and spacer length (bp)	Strains with recent inversions	Amino acid replacements due to inversion	Parental and strain sequences
*PIM1*	10, 3	YJM339	**S → G** **TCC → GGA**	CTCCAGAAGCtccGCTTCTGGAG CTCCAGAAGCggaGCTTCTGGAG
*REG2*	16, 5	YJM339	**K → D** **AAG → GAC,** **S → F** **TCT → TTT**	ACAGCGCCCTTGTTCAaagtcTGAACTAGATCCCTGT ACAGCGCCCTTGTTCAgacttTGAACAAGGGCGCTGT
*SYG1*	10, 4	JAY291	**Y → G** **TAC → GGT**, R → R CGG → AGG	ATCCAGCTCCtaccGGAGCTGGAT ATCCAGCTCCggtaGGAGCTGGAT
*AYR1*	10, 4	UWOPS05, YJM269	P → P CCT → CCA, **D → S** **GAT → TCA**	CTAATTTACCtgatGGTAAATTAG CTAATTTACCatcaGGTAAATTAG
*SPO75*	11, 2	D273-10B, CEN.PK, Y55	**D273-10B,** **CEN.PK:** **L → K,** **TTA → AAA** **Y55: K → L,** **AAA → TTA**	TGCCCGACGATttATCGTCGGGCA TGCCCGACGATaaATCGTCGGGCA
*ICT1*	10, 2	D273-10B, SEY6210, YJM789	L → L CTG → CTT, **K → Q** **AAG → CAG**	TGCAGGGCCTgaAGGCCCTGCA TGCAGGGCCTtcAGGCCCTGCA
*YPS1*	11, 3	UWOPS05	S → S TCT → TCG, **S → K** **TCG → AAG**	CCATACTGTTCttcGAACAGTATGG CCATACTGTTCgaaGAACAGTATGG
*ECM30*	10 , 2	W303	**D → S,** **GAT → TCT**	GGAGGACGCAgaTGCGTCCTCC GGAGGACGCAtcTGCGTCCTCC
*YBZ1*	10, 3	UWOPS05, Kyokai7, RedStar, CLIB215, L1528	**UWOPS05:** **E → F** **GAA → TTC** **Kyokai7, RedStar,** **CLIB215, L1528:** **F → E,** **TTC → GAA**	TGTCAATGCCttcGGCATTGACA TGTCAATGCCgaaGGCATTGACA
*TAT2*	11, 3	SEY6210	**E → F,** **GAA → TTC**	TTGGATTTGTAgaaTACAAATCCAA TTGGATTTGTAttcTACAAATCCAA

Nonsynonymous substitutions are shown in bold; the spacer are underlined.

UWOPS05 is a short for UWOPS05_217_3.

In nine out of the 10 genes with a spacer inversion, the inversion occurred in the case of previous homogenously perfect IR arms (only the spacer sequence changed). *SYG1* has 4 bp spacer inversion (Fig. 3). In this gene, a perfect IR arm of 10 bp was observed both in the derived JAY291 strain and in the ancestral form represented by S288c. The derived JAY291 strain had an inversion on the spacer, forming a 4 bp MNM. First, an intermolecular template switch occurring during the synthesis of the left arm, caused the 4 bp spacer inversion (Fig. 3b). Next, the nascent strand switched back to the original template (Fig. 3c). This inversion resulted in a Y46G replacement in the JAY291 strain (Fig. 3d).

**Figure 4:**
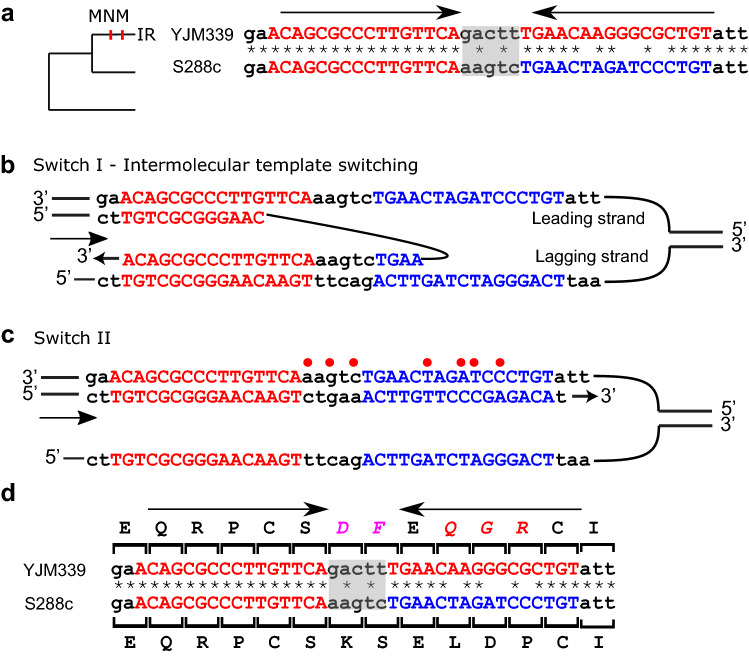
Intermolecular template switching causes arm homogenization and spacer inversion. (**a**) *REG2* has a perfect IR with an arm length of 16 bp in YHM339. The ancestral form represented by S288c has an arm length of 5 bp. The MNM occurred on the IR arm in the branch leading to YHM339 (shown as vertical lines on tree). (**b**) Intermolecular template switching, in which the first switch occurred during the synthesis of the first arm, was responsible for spacer inversion. (**c**) The second switch returned the nascent strand to the original template, resulting in an inverted spacer of YHM339 compared to S288c. (**d**) Seven substitutions were induced by this event (three on the spacer and four on the arm, two of which were part of a continuous MNM) resulting in five modified amino acids (KSELDP → DFEQRR, positions 206–211).

In only one event (*REG2*), did a spacer inversion occur together with conversion of an arm to form a perfect IR (Fig. 4). In this example, the ancestral form is represented by S288c, which has an IR with a 5 bp arm and a 5 bp spacer. This form evolved into a perfect IR with an arm length of 16 bp in the YJM339 strain. This event occurred through an intermolecular mechanism, similar to that presented in Fig. 3, and resulted in seven point mutations. Only two of these mutations were part of a continuous MNM, while the others were in a mutation cluster, 2 bp apart from each other (Fig. 4b, c). A total of five amino acid replacements were observed (Fig. 4d), the highest number of amino acid replacements resulting from template switching between IR arms that we identified in *S. cerevisiae* strains. Three amino acid replacements occurred on the arms (L208Q, D209G, P210R) and two occurred on the spacer (K205D, S206F).

In four of the genes, recent inversion of the spacer occurred independently in several strains, resulting in parallel evolution of this amino acid position. Spacer inversion occurred twice in *AYR1*, three times in *ICT1* and *SPO75*, and five times in *YBZ1*. In the genes *SPO75* and *YBZ1*, a flip inversion to two amino acid forms was observed.

**Figure 5:**
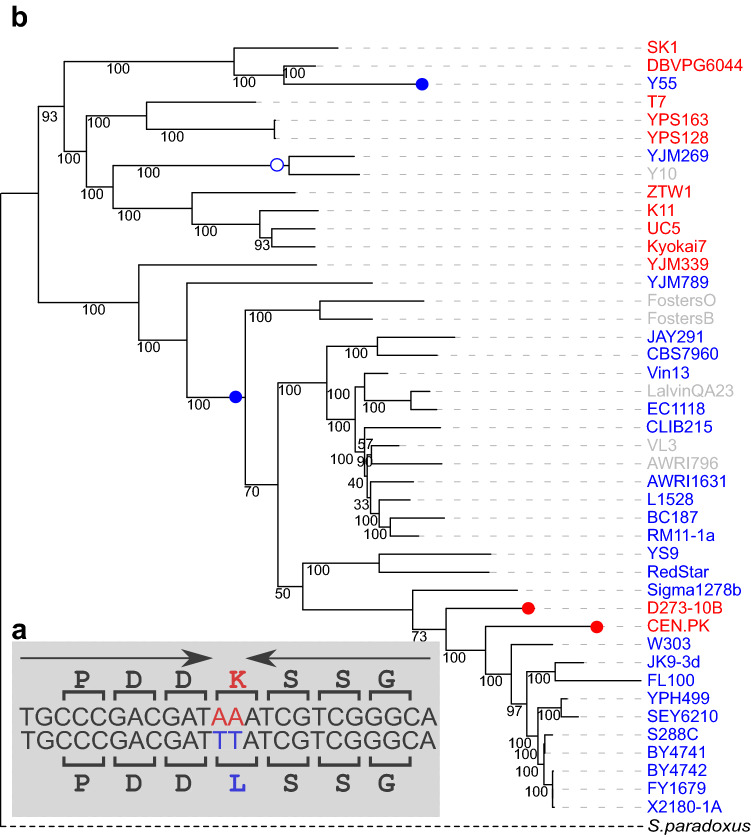
Spacer flip inversion in IR of *SPO75*. (**a**) IR with an arm length of 11 bp shows a perfect form in all *S. cerevisiae* strains. The 2 bp-spacer has either the ancestral form AA (red) or the derived form TT (blue) in positions 1 and 2 of codon 409 coding AAA (K) and TTA (L) respectively. (**b**) Tree presenting the template switching events. Strains in red have AA on the spacer, strains in blue have TT on the spacer, and strains in gray have indels in this region. *S. paradoxus* has AA in this locus but does not share the same IR. Three transitions are shown in full circles (two from L into K, shown in red, and one from K into L, shown in blue) on terminal branches. According to ancestral sequence reconstruction, there were also one to two reversal events from K to L on internal branches, one of which has only low support (empty circle). The best maximum likelihood tree reconstructed by RAxML using DNA sequences of 4304 orthologs and the ancestral sequence reconstruction of codon 409 by FASTML, are presented (see “Methods” section). Bootstrap values are shown on branches. Tree outgroup is not drawn to scale.

The two-base IR spacer appearing in *SPO75*, where perfect IRs with 11 bp arms appear in all *S. cerevisiae* strains (Fig. 5a), displays two forms: either the ancestral form AA (red), or the derived form TT (blue) in positions 1 and 2 of the alternative codons AAA (K) and TTA (L), respectively. As a result, Spo75 has one of two forms: K or L on codon 409. Three transitions were identified on the terminal branches (two from L to K, and one from K to L), two of which are on highly supported branches. In addition, ancestral sequence reconstruction indicated that one K-to-L reversal event occurred on an internal branch with a high support, and another with a low support (Fig. 5b). We concluded that spacer inversion is an event that can change forms within a short evolutionary time, usually on the basis of a perfect IR.

Spacer length is a key player in the formation of DNA structure and thus influences the frequency of template switching^60,61^. We examined whether spacer length of IRs associated with template switching differs from that of IRs that are not associated with template switching (Supplementary Fig. 1). The spacer length of IRs associated with MNMs with significant IR scores is shorter than that of IRs associated with MNMs with non-significant IR scores (one-tail Wilcoxon rank sum test *p* < 0.0011). Similarly, spacer length of IRs associated with MNMs with significant IR scores is also shorter than that of IRs with only single mutations on IR arms (one-tail Wilcoxon rank sum test *p* < 4.4171 × 10^−4^). Thus, spacers of IRs that undergo template switching to form MNMs are shorter than other IRs in coding genes.

### The nonsynonymous substitutions resulted from template switching

**Figure 6:**
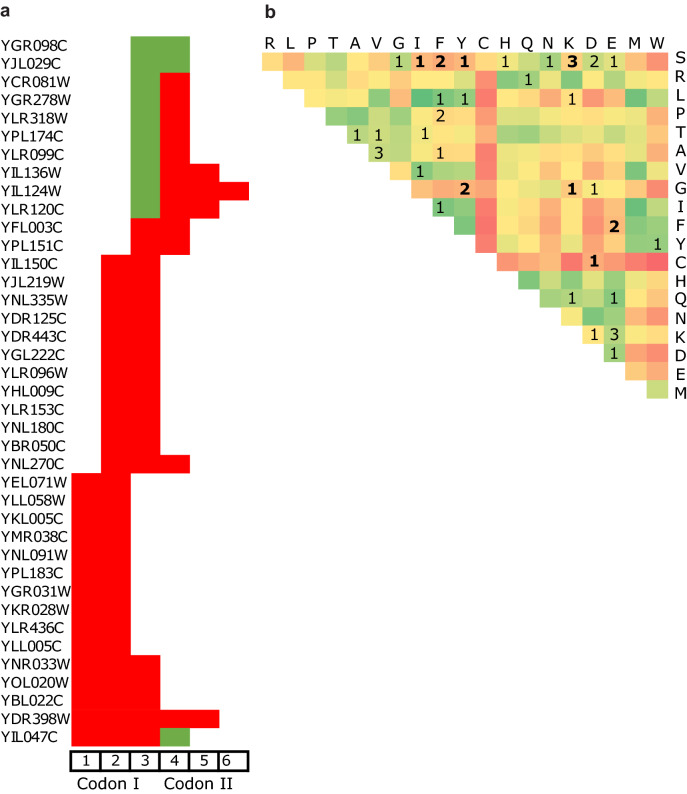
Effect of template switching on codons and amino acids. (**a**) Location of substitutions on two neighboring codons (six nucleotide positions). Synonymous substitutions are shown in green, and nonsynonymous substitutions are shown in red. For example, the MNM of gene YGR098C (top) are located in the 3rd position of the first codon and on the 1st position of the second codon. Both substitutions caused the synonymous changes shown in green. Similarly, YIL047C (bottom) has four neighboring substitutions spanning two codons. The first codon has three substitutions that replace the amino acid, shown in red, and the second codon has one synonymous substitution, shown in green. (**b**) The amino acid changes induced by MNMs in 39 proteins are shown; one event from its type per gene locus. The matrix is colored by Grantham's physicochemical distance table^62^ which has values of 5–215, with a mean distance of 100. Green represents similar amino acid pairs, while red represents distant amino acid pairs. Thirteen events had an amino acid with a physicochemical distance of 120 or higher (in bold).

MNMs associated with IRs can be located on a single codon or on two neighboring codons. Multiple differences in a single codon will change the amino acid (except for the rare case of serine). MNMs located on two codons will appear on the third position of the first codon and the first position of the second codon, and can result in a change between zero and two amino acids. Out of the 39 events associated with template switching, two resulted in synonymous substitutions only. In contrast, in 37 cases, at least one nonsynonymous substitution occurred (Fig. 6a). Out of these 37 events, in 24 genes, MNMs spanned a single codon and in 13 they spanned two codons. The number of transitions between the strains and their parental nodes in the template switching regions was higher than the number of transversions. The transition/transversion ratio was, however, smaller in template switching regions than in non-IR regions. As previously reported^57^, these nonsynonymous substitutions are prone to misidentification as positive selection sites. Indeed, arm and spacer MNMs in 26 out of the 37 genes were inaccurately estimated to have undergone positive selection, 15 of which showed very strong support (Supplementary Table 1).

Next, we sought to determine whether the amino acid replacements have the potential to alter the structure or function of proteins. Figure 6b presents all the amino acid changes we observed, against the background of a Grantham's physicochemical distances table^62^. This table is based on amino acid properties such as composition, polarity, and molecular volume. While most amino acid changes displayed low Grantham physicochemical distances, representing similar amino acid properties, 13 amino acid replacements displayed high Grantham's physicochemical distances (above 120).

We then used PredictSNP^63^, a classifier that combines several prediction tools, to identify the effect of mutations on protein structure and function. PredictSNP predicted that most amino acid changes had a neutral effect on protein function, while changes in six nonessential genes were considered non-neutral. These changes were L208Q in *REG2*, D98C in *DLD3*, G191K in *RTT10*, P50F in *OM45*, W127Y in *RHO5*, and G166Y in *CCS1*. The EggNOG database^64^ was then used to determine whether these novel amino acids are represented in other species during *Saccharomycotina* evolution. While the divergence time of *S. cerevisiae* and *S. paradoxus* is 4.0–5.8 mya, the origin of the budding yeast subphylum *Saccharomycotina* is 317–523 mya^65^. Five of the six genes had an orthologous group in EggNOG. In these genes, the alternative amino acid did not appear in *Saccharomycotina* (*OM45*, *RHO5*, *CCS1, DLD3,* and *RTT10*). In *RTT10*, the novel amino acid was, however, common when considering the entire *Ascomycota* phylum. Only two proteins of the genes *RHO5* and *DLD3* had an amino acid replacement in residues that show conservation throughout evolution. In conclusion, template switching introduced new nonsynonymous mutations into DNA coding genes during *S. cerevisiae* evolution. Most of the selected positions had physicochemical properties that were similar to those of their ancestral counterparts. In addition, amino acids with physicochemical properties distant from those of their ancestors, rarely occurred in conserved protein regions.

## Discussion

Template switching events have been previously reported in the context of loss of function in genes^34–37,51,52^. The question of how this affects normal gene evolution, however, has not been addressed. Given that template switching events involve multiple substitutions, our identification of template switching mediated by IRs in 39 *wild type S. cerevisiae* coding genes (~ 1% of the analyzed genes) was surprising. In most cases we identified in *S. cerevisiae* strains, template switching events yielded nonsynonymous substitutions. Most template switching events resulted in a single nonsynonymous substitution, although one extreme circumstance of five nearby amino acid replacements was also identified (Fig. 4). The influence of template switching on coding genes is probably even stronger than reported here, as we ignored cluster mutations that are not MNMs, indels, and MNMs occurring on internal branches.

This work focused on the effect that template switching between IR arms have on gene evolution. What we learned on the genetic mechanism of template switching was heavily affected by selection. We were, however, able to show that IRs involved in template switching have shorter spacers than other IRs, as previously reported^60,61^ (Supplementary Fig. 1). In our evolutionary data our findings were also influenced by the selection for short spacers in intermolecular template switching with spacer inversion (Table 2). This is because long inversion in proteins are unlikely to be neutral. There was a preference for spacer inversion when long IR arms were involved (Table 2, Figs. 3, 4).

In contrast to previously published data from bacteria in which template switching favored transversion over transition^66^ here we observed more transitions than transversions. The reason for this is probably due to selection over evolutionary time. Based on the structure of the genetic code, transitions are more likely than transversions to be synonymous, making transitions less often selected against than transversions^67,68^. IRs are unique since homogenization can occur when the nascent DNA strand folds and is used as a template. It is also of interest to further study the effect of other forms of repeats on protein evolution. Our preliminary results suggest that direct repeats can also promote the formation of MNMs.

The discovery that template switching between IR arms results in multiple events through evolution and, hence, in cryptic hotspots for parallel evolution, is an important finding. We note that identifying multiple template switching events (Figs. 2, 5) relies on the accuracy of the *S. cerevisiae* tree and the assumption that all genes within the *S. cerevisiae* lineage have the same evolutionary history. The reconstructed concatenated tree had a high bootstrap support.

Mutational hotspots have been previously suggested to be associated with parallel evolution at the amino acid level. For example, an elevated mutation rate at CpGs was shown to underlie hemoglobin adaptation in high-altitude Andean house wrens^69^. Template switching, however, was not previously considered a mechanism that contributes to parallel evolution in proteins. Here, we identified the reoccurrence of template switching over a short evolutionary period, yielding both arm homogenization and IR spacer inversion. Spacer inversions were reversible through sequential template switching events.

Codons with multiple changes between similar species can be the outcome of either a mechanism that simultaneously affects adjacent nucleotides, or of positive selection. Because substitutions are assumed to be independent, MNMs are sometimes considered evidence of multistep adaptive changes. Similarly, parallel evolution is usually considered evidence of adaptive selection. Such interpretations can be incorrect when the mutations occur together^54,55,57^. Indeed, when tested, 70% of what we recognized as template switching events on arms were inaccurately estimated as positive selection (Supplementary Table 1). Thus, our results suggest that template switching is a general mutagenic mechanism that causes MNMs, as well as parallel evolution, eliminating the need for adaptive explanations. Identification of the complex mechanisms that cause MNMs, such as template switching, error-prone translesion DNA synthesis^70^, gene conversion^71^, and probably other yet to be discovered processes, is essential in order to prevent overestimation of adaptive selection.

Although adaptive processes are not needed to explain MNMs caused by template switching and other complex mechanisms, this does not rule out the option that mutations formed by template switching can be a target for positive selection^54^. Most nonsynonymous mutations are eliminated by purifying selection and those that are fixed are usually replaced by physicochemically similar ones. However, nonsynonymous substitutions are still the ones with a small chance of improving function. By causing multiple nonsynonymous substitutions, template switching can, therefore, enable hopping between adaptive peaks without the crossing of low-fitness valleys in the adaptive landscape^54,72,73^. Based on the reported effect of synonymous mutations on RNA stability and protein translation efficiency^74^, even a combination of synonymous and nonsynonymous substitutions (Fig. 6) can promote a similar outcome.

## Methods

### Data collection and IR detection

The sequence of 6569 orthologous sets of coding DNA and protein sequences from 50 wild type *S. cerevisiae* strains were downloaded from the *Saccharomyces* Genome Database^75^. The *Saccharomyces paradoxus* ortholog of each gene was assigned based on the Fungal Orthogroups Repository^76^. Sequences with more than 20 *Ns* and sequences found in fewer than four strains were removed from the orthologous sets. These screenings resulted in a total of 4304 genes. For each orthologous group, we searched for IRs with an arm length of at least 7 bp and a spacer of up to 70 bp, in each of the available strains, using the EMBOSS palindrome package^77^. Each IR arm length was analyzed separately.

### Phylogeny

Each of the 4304 orthologous sets was aligned using MAFFT V3.705^78^ with default parameters. DNA MSAs were concatenated, and the best maximum likelihood (ML) tree was reconstructed by RAxML version 8.2.11^79^ under the GTR replacement matrix^80^, with among-site-rate-variation accounted for by assuming a discrete gamma distribution^81^ and with rapid bootstraps. This was the species tree used in this study.

### Ancestral tree reconstruction

Each orthologous set was also aligned by codon alignment. The phylogenetic species tree was pruned and used together with each orthologous codon MSA to estimate branch length and reconstruct ancestral codon sequences using FASTML^82^ with the M5 codon model^83^. In this step, orthologous sets with immature stop codon were eliminated, resulting in the 4252 orthologous sets that were used in this study.

### Identifying IRs that overlap MNMs

An MNM was defined when two or more adjacent substitutions were observed between a *S. cerevisiae* strain and its immediate parent node, as determined from FASTML ancestral reconstruction output. Thus, neighboring mutations mapping to two different branches were not identified as MNMs^54^. Insertions and deletions (indels) were not considered for MNM classification. IRs that mapped to a *S. cerevisiae* strain with overlapping MNMs in the terminal branch leading to this strain, were further analyzed. For each IR arm length, overlapping IRs on the same strain were excluded from the analysis. However, when an IR was fully nested in another IR, they were both analyzed.

### Elimination of false MSAs

To ensure MSA accuracy and avoid false MNMs, we used GUIDANCE2^84^ to score alignment regions with IRs in a codon model, using default parameters. We looked for IR regions with a cutoff higher than 0.95. In addition, codon MSA might not give the best MSA if insertion/deletions (indels) are not a multiple of three. We therefore used the similarity between nucleotide and codon comparisons in the IR region to identify high-quality MSAs. Codon and nucleotide pairwise alignments of the IR with a MNM and its sister taxa (plus a tail of 50 bp) were scored with a scoring matrix of match = 1, mismatch = − 1, and gap = 0. IRs were ignored if the difference between nucleotide and codon MSA scores was higher than 15, indicating a problematic codon MSA. Moreover, IR regions that included indels between the *S. cerevisiae* strain and their immediate parent node were ignored. Finally, to avoid mutation saturation, IR corresponding to branch lengths longer than 0.2 were ignored.

### Simulation

To determine the significance of association between IRs and MNMs, sequences were simulated along rooted phylogenetic trees using INDELible^85^ with the M5 model^83^. Each orthologous group codon MSA was simulated according to the FASTML phylogenetic tree using the inferred M5 evolutionary model parameters (kappa: transition/transversion ratio, and omega: dN/dS ratio) and PAML codeml^86^. In each simulation, the root was set to *S. paradoxus*. The sequence length for the INDELible simulation was set to four-five times the *S. paradoxus* sequence length in order to yield sufficient IRs with the exact arm length in each simulation. The length factor needed was selected based on the empirical evaluation of simulations. We continued the simulations until we obtained 100 MSAs in which the number of IRs in each was equal to or greater than the number of IRs in the real MSA. For each simulation in each orthologous set, the analysis performed for the real codon MSA was repeated. Each IR length was simulated separately.

### Control sequences

To correct for MNM enrichment that was not associated with IRs in the real MSA compared to the simulation, we searched for IR-less alignment segments of the same length as the IR. A control sequence was matched for each IR arm of the same length, in a non-IR region with the closest proximity to the IR. Thus, the number of IRs and control regions for each orthologous group was the same. If identical IRs were identified in multiple strains, the control regions were also chosen in the same positions and the same strains. Exact control analysis was also carried out for all simulations of the orthologous group.

### IR score

For each orthologous group and for each of its 100 simulations, the IR score was calculated as follows:

$$IR\;score = \frac{IRs\;with\;MNMs + 1}{{IRs\;without\;MNMs + 1}}{:}\frac{Controls\;with\;MNMs + 1}{{Controls\;without\;MNMs + 1}}$$

Namely, we calculated the ratio between the number of IRs with and without MNMs divided by the number of controls with and without MNMs. A pseudo count of 1 was added to all elements to prevent division by zero. The analysis was carried out separately for each IR arm length.

### Estimation of positive selection

Positive selection was estimated by PAML codeml^86^. MNMs that overlapped IRs were estimated with positive selection if Bayes empirical Bayes^87^ posterior probability under the positive selection model was above 0.5. Strong support for positive selection was identified if posterior probability under the positive selection model was above 0.95.

### Novelty of amino acid replacements

PredictSNP^63^ classifier with default parameters was used to identify amino acid replacements with a potential affect of protein structure. Proteins with a predicted affect on structure were manually inspected in the EggNOG database of orthology relationships^64^. For each protein we identified whether the replaced amino acid was represented in the MSA of *Saccharomycotina*^64^.

## Supplementary Information

Supplementary Information.
